# Cartilage Oligomeric Matrix Protein–Derived Peptides Secreted by Cartilage Do Not Induce Responses Commonly Observed during Osteoarthritis

**DOI:** 10.1177/1947603520961170

**Published:** 2020-09-29

**Authors:** Enrique Andrés Sastre, Frank Zaucke, Janneke Witte-Bouma, Gerjo J.V.M van Osch, Eric Farrell

**Affiliations:** 1 Department of Oral and Maxillofacial Surgery, Erasmus MC, University Medical Center Rotterdam, the Netherlands; 2 Dr. Rolf Schwiete Research Unit for Osteoarthritis, Orthopaedic University Hospital Friedrichsheim, Frankfurt, Germany; 3 Department of Orthopaedics and Department of Otorhinolaryngology, Erasmus MC University Medical Center, Rotterdam, the Netherlands

**Keywords:** cartilage oligomeric matrix protein, matrikine, synovium, endothelial cells, transforming growth factor-β

## Abstract

**Objective:**

To evaluate if 3 peptides derived from the cartilage oligomeric matrix protein (COMP), which wounded zones of cartilage secrete into synovial fluid, possess biological activity and might therefore be involved in the regulation of specific aspects of joint regeneration.

**Methods:**

The 3 peptides were produced by chemical synthesis and then tested *in vitro* for known functions of the COMP C-terminal domain from which they derive, and which are involved in osteoarthritis: transforming growth factor-β (TGF-β) signaling, vascular homeostasis, and inflammation. *Results*. None of the peptides affected the gene expression of *COMP* in osteochondral progenitor cells (*P* > 0.05). We observed no effects on the vascularization potential of endothelial cells (*P* > 0.05). In cultured synovium explants, no differences on the expression of catabolic enzymes or proinflammatory cytokines were found when peptides were added (*P* > 0.05).

**Discussion and Conclusions:**

The 3 peptides tested do not regulate TGF-β signaling, angiogenesis and vascular tube formation, or synovial inflammation *in vitro* and therefore most likely do not play a major role in the disease process.

## Introduction

Osteoarthritis (OA) is the most prevalent chronic degenerative joint disease. It affects all tissues of the joint, including the cartilage, synovium, vascular system, and subchondral bone.^1^ During OA, the cartilage matrix experiences degradative changes, resulting in the release of proteins and their fragments into the synovial fluid. OA can be diagnosed by detecting these degradation products in synovial fluid and serum, as biomarkers of the disease stage. For this reason, extensive research has focused on looking for more specific and reliable biomarkers associated with OA, in particular novel neoepitopes produced during proteolysis.^2^ However, little is known about possible biochemical functions that these fragments may still play during the onset of the disease and its progression. Bioactive fragments of extracellular matrix proteins, known as matrikines and matricryptins, are produced by limited proteolysis and can regulate a wide variety of cellular functions such as cell adhesion, migration, proliferation, angiogenesis, or apoptosis.^3^ Multiple matricellular proteins give rise to matricryptins. For example, the matricryptin PEX derives from the C-terminal domain of the matrix metalloproteinase-2 (MMP-2) and inhibits MMP-2 enzymatic activity in a negative feedback loop, blocking in turn angiogenesis.^4^ Other matricryptins can promote catabolic and proinflammatory responses, such as the aggrecan 32-mer fragment does in cartilage^5^ or fibronectin fragments do on monocytes and cartilage.^6,7^

After joint injury and in early stages of osteoarthritis, increased amounts of cartilage oligomeric matrix protein (COMP) fragments are released into synovial fluid.^8^ COMP, which belongs to the family of thrombospondins, is currently being used as a biomarker of joint destruction,^9^ as its concentration correlates with both the severity of the disease as well as with the number of affected joints. An abstract published by Calamia *et al*.^10^ in 2016 identified 3 specific peptides derived from COMP that wounded zones of cartilage release into synovial fluid. The 3 peptides reported originate from the multifunctional C-terminal domain of COMP, which plays diverse roles in cartilage homeostasis,^11^ inflammation,^12^ and transforming growth factor-β (TGF-β) signaling.^13,14^ Previous research found that full-length COMP was unable to modify the expression of proinflammatory markers in cartilage,^15^ unlike its binding partner MATN3 and its fragments.^16^ However, we hypothesized that those COMP fragments released by cartilage could have an OA-relevant biological function.

In this work, we asked if any of those 3 peptides is involved in responses associated to osteoarthritic joint disease; for example, by affecting vascularization, by modulating downstream TGF-β signaling or by altering the expression of synovial catabolic enzymes.

## Methods

### Peptides

Three peptides were produced by chemical synthesis (Peptide2.0, Chantilly, VA, USA), with the sequences ^631^AEPGIQLKAV (peptide 1) ^642^SSTGPGEQLRNA (peptide 2) and ^553^VLNQGREIVQT (peptide 3), reconstituted in dimethyl sulfoxide (DMSO) and stored at −80 °C. Phosphate buffered saline (PBS) was used to adjust the concentration and equivalent DMSO dilutions were used as controls on the experiments. To screen for the possible functions of the peptides, we selected a single concentration of 100 nM for most of the assays. We based our choice on the maximum amount of peptides that 10 μg/mL of the parent protein COMP would release if COMP was completely cleaved, as COMP demonstrates bioactive effects *in vitro* at this concentration^17,18^ and COMP is present in this concentration range in human serum.^19^

### Cell Culture

Human umbilical vein endothelial cells (HUVECs) were purchased from Lonza, and expanded in endothelial growth media (EGM-2), containing 2% fetal bovine serum (FBS), 5 ng/mL epidermal growth factor (EGF), 10 ng/ml basic fibroblast growth factor (bFGF), 20 ng/mL insulin-like growth factor (IGF), 0.5 ng/mL vascular endothelial growth factor (VEGF) 165, 1 µg/mL l-ascorbic acid 2-phosphate, 22.5 µg/mL heparin, and 0.2 µg/mL hydrocortisone (all from Promocell via Bioconnect, Huissen, the Netherlands). Peptide testing was carried on endothelial basal media (EBM-2), which did not contain growth factors or supplements (Lonza, Geleen, the Netherlands).

Osteochondroprogenitor cells were isolated from leftover iliac crest bone chip material obtained from 4 pediatric patients undergoing alveolar bone graft surgery (following parental consent and approval of medical ethics committee of Erasmus MC: MEC-2014-16; age 9-13 years). Cells were expanded in α-minimum essential medium (αMEM; Gibco, Bleiswijk, the Netherlands) containing 10% FBS (Gibco, Bleiswijk, the Netherlands) and supplemented with 50 µg/mL gentamycin (Gibco, Bleiswijk, the Netherlands), 1.5 µg/mL amphotericin B (Gibco, Bleiswijk, the Netherlands), 25 µg/mL l-ascorbic acid 2-phosphate (Sigma, Zwijndrecht, the Netherlands), and 1 ng/mL FGF-2 (Bio-Rad via Bioconnect, Huissen, the Netherlands) in a humidified atmosphere at 37 °C and 5% CO_2_. Cells from passages 4 and 5 were used. For the following cell stimulation experiments, peptides were incorporated to αMEM supplemented with 50 µg/mL gentamycin, 1.5 µg/mL amphotericin B, and 1.25 mg/mL bovine serum albumin (BSA; product code 1002759876, Sigma, Zwijndrecht, the Netherlands). For those experiments involving cell stimulation with the peptides in combination with TGF-β, a dose of 0.1 ng/mL of TGF-β3 (R&D, Abingdon, UK) was added to the stimulation media. This dose of TGF-β3 was first determined experimentally and corresponded to the half-effective dose to upregulate the expression of *COMP* in the osteochondroprogenitor cells (data not shown), which corresponded to a molar ratio of TGF-β3:peptide of 1:25.

### Migration Assay

Migration was assessed with modified Boyden chambers (polyethylene terephthalate cell culture inserts with 8 µm pore size) (Corning, Durham, NC, USA). In brief, 5 × 10^4^ HUVECs were seeded on the upper insert membrane in EBM-2 and allowed to migrate toward the lower chamber containing EBM-2 and the peptides for 10 hours at 37 °C. In order to ensure the ability of the cells to migrate, EGM-2 was loaded in parallel as a positive control and the basal medium EBM-2 was used as a negative control. Then, the cells on the membrane were fixed with 4% formalin and cells on the upper surface were removed with a cotton swab, followed by 5-minute DAPI (4′,6-diamidino-2-phenylindole) staining. Migrated cells on each membrane were imaged with a fluorescence microscope (Zeiss Axiovert 200M) in 5 random fields of 1.51 mm^2^ each, automatically counted using ImageJ software, and the average counts per membrane expressed as cells/mm^2^.

### Proliferation Assay

The number of HUVECs that proliferated after 24 hours was investigated with the EdU cell proliferation kit (Baseclik, Neuried, Germany). First, 7,500 cells/cm^2^ were seeded in EGM-2 and allowed to attach overnight. Then, cell cycles were synchronized by substituting the media with EBM-2 supplemented with 1.25 mg/mL BSA for 8 hours. Next, cells were stimulated with the peptides in combination with EdU 10 µM in EBM-2. EBM-2 alone was used as a negative control for proliferation, and EGM-2 was used as a positive control. After 24 hours, cells were fixed with 4% formalin and stained according to the manufacturer’s kit protocol. Finally, positive cells were imaged with a fluorescence microscope (Zeiss Axiovert 200M) in 5 random fields of 1.51 mm^2^ each. Total nuclei (DAPI) and EdU+ nuclei were automatically counted using ImageJ software. Percentage of proliferated cells per field was calculated as the number of EdU+ nuclei divided by total nuclei. Then, total proliferation per membrane was calculated as the average proliferation of its 5 fields.

### Tube Formation Assay

Fifty microliters of Geltrex LDEV-Free Reduced Growth Factor Basement Membrane Matrix (Fischer Scientific, Landsmeer, the Netherlands) were allowed to polymerize for 30 minutes at 37 °C on a 96-well plate. Then, HUVECs were resuspended in EBM-2 containing the peptides seeded at a density of 2 × 10^4^ cells per cm^2^ and incubated at 37 °C. EGM-2 and EBM-2 were used as positive and negative controls, respectively. After 24 hours, 5 random images of the tubes formed (1.9 mm^2^ each) were taken using an inverted microscope. Automatic measurements of the tubes were performed with the Angiogenesis Analyzer plugin for ImageJ to determine the average number of nodes per condition.

### Viability Assay

Osteochondroprogenitor cellular viability in presence of the peptides was assessed with the colorimetric MTT assay. This assay relies on the metabolic reduction of the tetrazolium dye MTT to formazan, which has a purple color. A total of 24,000 cells/cm^2^ from one single donor were seeded in expansion media. Next day, medium was replaced by αMEM supplemented with 50 µg/mL gentamycin, 1.5 µg/mL amphotericin B, 1.25 mg/mL BSA, and with different peptides concentrations up to 1 µM for 24 hours. MTT was added into the media at a final concentration of 0.9 mM during the 3 last hours. After a PBS wash, precipitated colorant was extracted with absolute ethanol. Absorbance was measured on a spectrophotometer (VersaMax, Molecular Devices), as A_570_ − A_670_. Finally, viability was calculated as absorbance relative to the untreated condition.

### Synovial Explant Culture

Synovial explants were obtained from leftover material from 4 patients undergoing total knee replacement surgery, both male and female and ranging from 67 to 77 years old, at the hospitals of Erasmus MC, Rotterdam and Reinier de Graaf Gasthuis, Delft (the Netherlands). The patients gave implicit consent as stated by guidelines of the Federation of Biomedical Scientific Societies (www.federa.org) and with approval of the local ethical committee at Erasmus MC (MEC-2004-322). Explants were cut in pieces of similar size and washed with saline solution (0.90% w/v of NaCl). Then, they were cultured for 24 hours in low-glucose DMEM (Dulbecco’s modified Eagle medium) containing 1:100 v/v insulin-transferrin-selenium (ITS+; Corning, Amsterdam, the Netherlands), 50 µg/mL gentamycin, and 1.5 µg/mL amphotericin B. Peptides were supplemented twice during the following 48 hours. Explants were snap frozen and stored at −80 °C for further analyses.

### Gene Expression

Frozen samples were microdismembrated and total RNA was isolated using the RNeasy Plus Micro kit (Qiagen-Benelux, Venlo, the Netherlands). Three hundred nanograms RNA were reverse transcribed to cDNA using the RevertAid First Strand cDNA Synthesis Kit (Thermo Scientific, Bleiswijk, the Netherlands). mRNA expression was measured for *COMP, B2M, TNFA, IL6*, and *UBC* with qPCR Mastermix Plus for SYBR Green I (Eurogentec, Nederland B.V., Maastricht, the Netherlands). For *ADAMTS4, ADAMTS5, GAPDH, MMP1, MMP3*, and *MMP13*, TaqMan Master Mix (ABI, Branchburg, NJ, USA) was used. Primer sequences were as follows:

SYBR probes.
*COMP* fw: 5′-CCCCAATGAAAAGGACAACTGC-3′; rv: 5′-GTCCTTTTGGTCGTCGTTCTTC-3′
*B2M* fw: 5′-TGCTCGCGCTACTCTCTCTTT-3′; rv: 5′-TCTGCTGGATGACGTGAGTAAAC-3′
*TNFA* fw: 5′-GCCGCATCGCCGTCTCCTAC-3′; rv: 5′-GCCGCATCGCCGTCTCCTAC-3′
*IL6* fw: 5′-TCGAGCCCACCGGGAACGAA-3′; rv: 5′-GCAGGGAGGGCAGCAGGCAA-3′
*UBC* fw: 5′-ATTTGGGTCGCGGTTCTTG-3′; rv: 5′-TGCCTTGACATTCTCGATGGT-3′
TaqMan probes.
*ADAMTS4* fw: 5′-CAAGGTCCCATGTGCAACGT-3′; rv: 5′-CATCTGCCACCACCAGTGTCT-3′;
  probe: FAM-5′-CCGAAGAGCCAAGCGCTTTGCTTC-3′-TAMRA
*ADAMTS5* fw: 5′-TGTCCTGCCAGCGGATGT-3′; rv: 5′-ACGGAATTACTGTACGGCCTACA-3′;
  probe: FAM-5′-TTCTCCAAAGGTGACCGATGGCACTG-3′-TAMRA
*GAPDH* fw: 5′- ATGGGGAAGGTGAAGGTCG-3′; rv: 5′-TAAAAGCAGCCCTGGTGACC-3′;
  probe: FAM-5′-CGCCCAATACGACCAAATCCGTTGAC-3′-TAMRA
*MMP1* fw: 5′-CTCAATTTCACTTCTGTTTTCTG-3′; rv: 5′-CATCTCTGTCGGCAAATTCGT-3′;
  probe: FAM-5′-CACAACTGCCAAATGGGCTTGAAGC-3′-TAMRA
*MMP3* fw: 5′-TTTTGGCCATCTCTTCCTTCA-3′; rv: 5′-TGTGGATGCCTCTTGGGTATC-3′;
  probe: FAM-5′-AACTTCATATGCGGCATCCACGCC-3′-TAMRA
*MMP13* fw: 5′-AAGGAGCATGGCGACTTCT-3′; rv: 5′-TGGCCCAGGAGGAAAAGC-3′;
  probe: FAM-5′-CCCTCTGGCCTGCGGCTCA-3′-TAMRA

Data were analyzed using the Livak method (2^−ΔΔCT^). For the set of experiments of osteochondroprogenitor cells, the reference gene used was *GAPDH*. For the set of experiments using synovial explants, normalization was based on the BestKeeper Index (BKI) using the genes *GAPDH, UBC*, and *B2M*. Gene expression was expressed relative to the untreated condition.

### Statistical Analysis

Differences between treatments were assessed with 1-way analysis of variance, 2-sided with Dunnett’s *post hoc* test. For those experiments involving multiple donor samples, data were analyzed using the linear mix model with Bonferroni correction. In both cases, normality of the data was assumed. Statistics were performed with IBM SPSS Statistics (version 25.0.0.1 for Windows, IBM Corp., Armonk, NY, USA), and graphs were created using GraphPad Prism (version 6.1 for Windows, GraphPad Software, La Jolla, CA, USA).

## Results

### None of the Peptides Were Able to Modify the Gene Expression of *COMP*

**Figure 1.**
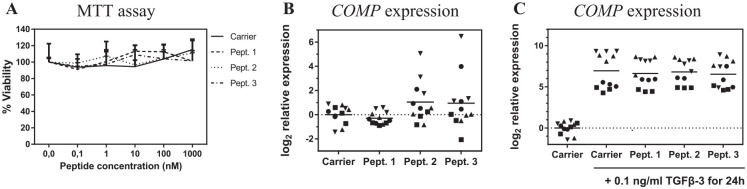
Effects of the 3 peptides on bone marrow osteochondroprogenitor cell viability and *COMP* expression. (**A**) Viability assay. Cells were incubated for 24 hours in combination with the peptides, then viability was quantified by the MTT assay where the signal was normalized to the untreated cells (*n* = 1 donor by quadruplicate) and each peptide concentration was compared with the equivalent carrier concentration; bars represent SD. (**B**, **C**) Gene expression of *COMP* (*n* = 4 donors by triplicate) was measured by qPCR (quantitative polymerase chain reaction) after 24 hours on incubation with the peptides at 100 nM (**B**) or 100 nM peptides plus 0.1 ng/mL TGF-β3 (**C**). In both cases, all treated conditions were normalized to *GAPDH*, and relative to the dimethyl sulfoxide (DMSO) only condition; each donor is represented by a different symbol. Grand mean is represented.

TGF-β can trigger the expression of *COMP*,^20^ and the resulting COMP protein can further enhance TGF-β activity in a positive feedback loop. Because the 3 peptides derive from the COMP C-terminal domain, which is responsible for the binding to TGF-β,^13^ we hypothesized that the peptides could have a modulatory activity over this COMP-TGF-β-*COMP* feedback loop. Therefore, we investigated whether any of the peptides could modulate the expression of their parental gene *COMP*. For this, we used bone marrow osteochondroprogenitor stem cells, which have a moderate ability to differentiate and to repair damaged articular cartilage by depositing fibrocartilage.^21^ After confirming that the peptides did not affect cellular viability (**Fig. 1A**), we evaluated whether any of the peptides could upregulate the expression of *COMP* at the mRNA level. Consequently, cells were stimulated with the peptides at 100 nM and *COMP* expression was assessed by qPCR. However, no significant differences were found in *COMP* expression levels (**Fig. 1B**). Then, we asked if any of the peptides could modulate COMP production when TGF-β signaling was triggered. Accordingly, *COMP* expression was measured after 24 hours of peptide stimulation in presence of TGF-β3. Despite *COMP* was being upregulated by TGF-β in a donor-dependent manner, no differences in *COMP* expression were found in the presence or absence of the peptides.

### None of the Peptides Affected Vascular Tube Formation *In Vitro*

**Figure 2.**
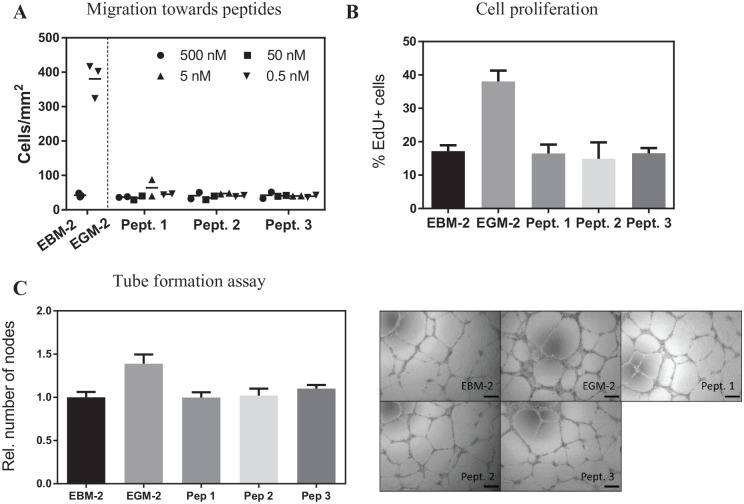
Assays to test the peptides influence on vascularization potential by endothelial cells (HUVECs). (**A**) Migration assay. Cells were seeded on a microporous membrane and allowed to migrate towards a compartment with different concentrations of each of the peptides (*n* = 2/concentration). (**B**) Proliferation assay. Cells were incubated for 8 hours in basal medium followed by a 24-hour exposure to 100 nM peptides in basal medium (*n* = 3 replicates). (**C**) Cells were seeded on GeltrexTM in combination with the peptides at 100 nM and allowed to form a tubular network for 24 hours. Then, nodes formed were quantified (*n* = 3 replicates). Scale bar = 200 µm. In all experiments, both basal and growth media contained the same concentration of vehicle (dimethyl sulfoxide [DMSO]), and growth media was used as a positive control. EBM-2, endothelial basal media 2; EGM-2, endothelial growth media 2. −(EBM-2), +(EGM-2). The bars represent the mean (**A**) and the mean ± SD (**B**, **C**). All samples were compared with the untreated condition.

During osteoarthritis, increased vascular remodeling is seen in the synovium^22^ and in the cartilage, which is normally avascular in healthy adult joints.^23^ In order to study if the peptides would be capable of modulating vascularization, we performed different *in vitro* assays. First, we allowed endothelial cells (HUVECs) to migrate in a modified Boyden chamber assay with the peptides, ranging in concentrations from 0.5 to 500 nM, and we observed that none of the peptides were chemokinetic for endothelial cells at the concentrations tested (**Fig. 2A**). Next, we asked if the peptides could stimulate endothelial cell proliferation, as it is one of the common responses to angiogenic stimuli. For this purpose, cells were stimulated for 24 hours in a media containing the nucleoside analogue EdU, which labeled the replicating cells. The peptides did not significantly increase the number of proliferating cells (**Fig. 2B**). Last, we performed a tube formation assay with HUVEC on a basement membrane coated plate stimulated with the peptides for 24 hours. In this case, peptides did not influence network formation (**Fig. 2C**).

### None of the Peptides Altered Gene Expression of Synovial Catabolic Enzymes or Inflammatory Mediators

**Figure 3.**
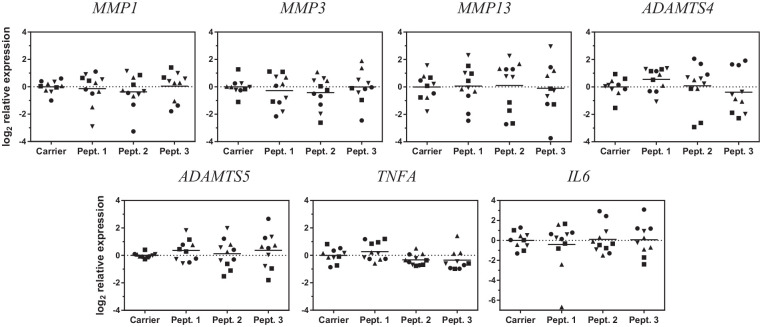
Effect of the peptides on gene expression of proteolytic enzymes in synovium. Synovial explants were treated for 48 hours with the peptides and gene expression of different proteolytic enzymes was measured by quantitative polymerase chain reaction (qPCR). Each gene expression measurement was first normalised to the BestKeeper Index (BKI). Each condition is relative to the carrier control, to which is compared; each donor is represented by a different symbol (*n* = 4 donors in triplicate, one of them in duplicate) and grand mean is represented.

As the peptides were found in synovial fluid, we asked if they could trigger an inflammatory response on the synovium. For this, synovial explants from patients undergoing total knee replacement surgery were cultured with 100 nM of the peptides or with the carrier (DMSO) for 48 h, and gene expression of *MMP1, -3, -13* and *ADAMTS-4* and -*5*—proteases known to degrade extracellular matrix in osteoarthritis—was analyzed. Gene expression of none of the proteases analyzed was affected by the presence of the peptides (**Fig. 3**). *IL6* and *TNFA*, which are potent regulators of catabolic processes in chondrocytes and synovial cells, were also unaffected by exposure to the peptides.

## Discussion and Conclusions

Our work has focused on the search for novel bioactive fragments of COMP, one of the main extracellular matrix proteins of the cartilage which is often used as a biomarker for OA. For this, we tested if any of the three COMP-derived peptides reported by Calamia *et al*.^10^ in 2016 could affect specific aspects related to OA. We concluded that those 3 peptides are unlikely to be involved in the expression of *COMP*, vascularization, or synovial expression of extracellular matrix proteases at the concentrations and time frames studied.

Identification of novel matrikines and matricryptins is challenging, as their functions can be similar, opposite, or completely different to their parent protein. During OA, increased amounts of COMP are released^24^ and increased concentrations of monomeric forms of COMP as well as smaller fragments appear as a result of proteolytic activity.^25^ In a recent study, we showed that in the absence of its oligomerization domain, which is required to form pentameric structures, COMP’s angiogenic-related functions disappear.^26^ Matrikines and matricryptins, however, have been shown to have different biological functions to the protein from which they are derived, leading us to assess the effects of these identified peptides on processes crucial for the onset and development of OA.

The 3 peptides derive from COMP’s C-terminal domain: ^631^AEPGIQLKAV (peptide 1), ^642^SSTGPGEQLRNA (peptide 2), and ^553^VLNQGREIVQT (peptide 3) and are well conserved across species. Peptides 1 and 2 are completely identical among mammals, while ^558^R in peptide 3 is exclusive for primates instead of the ^558^M found among other mammals. Interestingly, peptides with similar sequences can be potentially released from other proteins of the thrombospondin family, due to their high similarities. Although it is not clear which are the proteases responsible to produce these peptides, they might be related to OA-relevant proteases. ADAMTS-4 could be responsible for the production of ^553^V cleavage on peptide 3.^27^ These peptides are not likely to be involved in PSACH/MED, as none of the closest COMP mutations fall on the peptides ^529^T, ^585^T, and ^718^R.^28-30^ The 3 peptides span sequences where secondary structures initiate: beta sheets for peptides 1 and 3 and alpha helix for peptide 2. However, it is possible that the proteolytic events that release the peptides from the protein results on a peptide’s secondary structure different to the one in the parent protein. Our experiments were designed based on the known functions of the protein domain where the peptides are derived from. The main limitations for this study were that we could not account for the possible interactions between the peptides and other factors. In our screening approach, we assumed that cellular responses would be proportional to the peptide concentration, and that our selected single dose would be the highest possible within the physiological range. We cannot exclude, however, that other doses or experimental durations (though optimized previously) could have had an effect on the processes. Also, we could not account for the possibility that the synovial explants used may have already encountered the peptides *in vivo*, which could have led to some level of desensitization. In addition, we observed a high variability between donors and between explants of the same donor. This could be explained by the different degrees of inflammation and synovial damage of the patients, which could turn a patient unresponsive to our testing stimuli. In order to reduce interindividual variability, it would be advisable for future experiments to seek for sources of healthy synovium. Last, because matrikines and matricryptins may play different roles to their parent proteins, we cannot exclude their involvement on other processes in OA than those analyzed. Following this research, Calamia *et al*.^31^ identified more peptides derived from COMP, in parallel to multiple peptides derived from other matricellular proteins. For future preselection of candidates, it might be advisable to prioritize those matrikines and matricryptins either known or strongly suspected to be biomarkers of diseases, in particular if containing certain motifs such as RGD or known interaction sites for growth factors. The reason is that, because diseased individuals possess those molecules in concentrations different than healthy individuals, those molecules are more likely to be actively involved into the pathogenesis of the disease, either by playing detrimental roles or by stimulating compensatory mechanisms.
